# A Mild and Facile One-Pot Synthesis of *N*-Methyl-3-Acyl-Pyrroles

**DOI:** 10.3390/molecules15052972

**Published:** 2010-04-27

**Authors:** Hassan Valizadeh, Ashraf Fakhari

**Affiliations:** Department of Chemistry, Faculty of Sciences, Azarbaijan University of Tarbiat Moallem, Tabriz, Iran

**Keywords:** 3-acylpyrrole, multicomponent reaction, dimethylacetylene dicarboxylate

## Abstract

*N*-Methyl-3-acylpyrroles were synthesized via a multicomponent reaction of dimethylacetylene dicarboxylate (DMAD), *N*-methylhydroxylamine and acylchlorides in the presence of KHCO_3_. For comparison both conventional and microwave protocols were examined in this procedure. The reaction is clean and gives the products in good to excellent yields under conventional heating conditions at 40 ºC in anhydrous dichloromethane.

## 1. Introduction

Pyrrole is one of the most important heterocyclic compounds, having become increasingly important in medicinal chemistry and organic synthesis [1,2,3,4,5]. Some of the recently isolated pyrrole-containing marine natural products have been found to exhibit considerable cytotoxicity and function as multi drug resistant (MDR) reversal agents. Many of these biologically active compounds have emerged as chemotherapeutic agents [6]. Heterocyclic compounds containing a 3-acylpyrrole fragment are of interest for making new pharmacological preparations. For example, the cannabinoid activity of 1-alkyl-3-(naphthoyl)pyrroles [7] and 1-alkyl-3-(naphthoyl)indoles [8] is known, as is the antibiotic activity of verrucarin E (3-acetyl-4-hydroxymethylpyrrole) isolated from *Myrothecium verrucaria* [9]. 3-Acylpyrroles and other 3-substituted pyrroles obtained from them are precursors of liquid crystal materials [10] and polypyrroles [11], possessing high electrical conductivity in comparison with their 1-substituted analogs [12]. Such conjugated polymers may be used, for example, to construct gas sensors [13], and also sensors capable of distinguishing DNA molecules [14]. Many methods for the synthesis of diversely substituted pyrroles have been developed [15]. Conjugate addition reactions [16], transition metal-mediated reactions [17], reductive couplings [18], aza-Wittig reactions [19], and other multistep operations [20] have all been performed for the synthesis of pyrroles. Despite these huge developments, the Paal-Knorr [21] reaction is still considered to be the most attractive method for the synthesis of pyrroles. In the present work, we describe a simple method for the KHCO_3_-catalyzed synthesis of 3-acylpyrroles in good yields starting from commercially available DMAD, *N*-methylhydroxylamine and various acylchlorides.

## 2. Results and Discussion

Our research group has reported several studies directed toward the synthesis of some heterocyclic compounds under solvent-free conditions [22,23,24,25,26,27]. Because of the biological activities of pyrrole derivatives, we became interested in the one-pot synthesis of *N*-methyl-3-acylpyrroles under mild and solvent-free conditions.

**Scheme 1:**
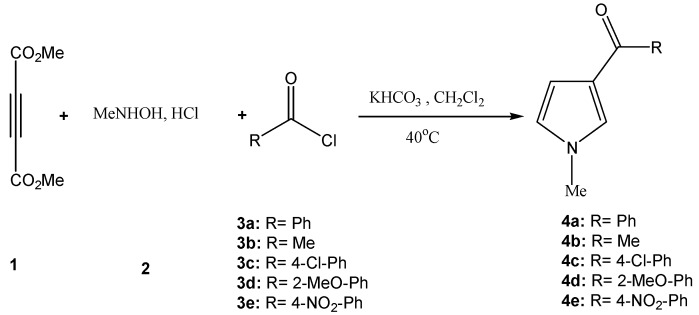
One-pot synthesis of *N*-methyl-3-acylpyrroles.

Electrophilic substitution in pyrrole occurs predominantly at the C_2_-position [28,29,30]. Thus, the investigation of efficient methods for preparing C_3_-substituted pyrroles is one of the important goals in pyrrole chemistry because of their frequent use for obtaining various biologically active compounds like porphyrins. For the substitution on β-(C_3_) position, Friedel-Crafts acylation or alkylation on pyrrole bearing an electron withdrawing substituent at C_2_ [31,32] or N_1_ [33,34] has been widely investigated. Direct Friedel-Crafts acylation on 2,5-dimethylpyrrole also gives 3,4-symmetric acyl compounds, but normally the yields are very low ( < 5%) when no electron withdrawing groups are on the substituted pyrroles [18]. Very recently we prepared pentasubstituted pyrroles via the reaction of DMAD with *N*-methylhydroxylamine under mild solvent-free conditions, so we studied the possibility of synthesizing *N*-methyl-3-acylpyrroles **4a-e** using a multicomponent reaction in anhydrous dichloromethane of DMAD (**1**), *N*-methylhydroxylamine (**2**) and various acylchlorides **3a-e** in the presence of KHCO_3_ (Scheme 1).

We tried several different reaction conditions under microwave irradiation and conventional heating, and found that the reaction took place efficiently and in good yield at 40 ºC in CH_2_Cl_2_. Firstly, benzoyl chloride (**3a**) was chosen as a model for the reaction with DMAD and *N*-methylhydroxylamine to optimize reaction conditions. It was observed that a low yield of **4a** was obtained in the absence of KHCO_3_ when the reaction was stirred at room temperature for 10 h. The reaction in the presence of the KHCO_3_ at room temperature in CH_2_Cl_2_, afforded the corresponding product in 61% yield (Table 1, entry 1). The reaction was also examined at 40 ºC and found to be faster and to give higher yield (81%) than at room temperature (Table 1, entry 2). The procedure was examined under microwave-assisted conditions and found that the yield of product was very low in comparison with conventional heating in anhydrous CH_2_Cl_2_ (Table 1, entries 7-8). In another experiment, for the same reaction, several common solvents were examined in the presence of KHCO_3_at 40 ºC. The results showed that the reaction in CH_2_Cl_2_ resulted in higher yields in comparison with the other tested solvents (Table 1, entries 2-5). There is no significant difference in the results using different bases such as Na_2_CO_3_, K_2_CO_3_, KHCO_3_, NH_4_OAc, NaOH, and NH_4_Cl as the catalyst in this procedure. The reaction of enamine, derived from addition of an *N*-methylhydroxylamine to DMAD, and benzoylchloride proceeds by a smooth 1:1:1 addition reaction in anhydrous dichloromethane at 40ºC to produce N-mehyl-3-benzoylpyrrole (**4a**) in 81% yield.

**Table 1:** Study of the synthesis of 3-benzoylpyrrole **4a** under different conditions.

Entry	Solvent/Solid support	Mode of heating	Temperature (ºC)	Time (h)	Yield^a^(%)
1	CH_2_Cl_2_	Conventional	25	3	61
2	CH_2_Cl_2_	Conventional	40	2.5	81
3	CHCl_3_	Conventional	40	3	63
4	Dioxane	Conventional	40	2.5	60
5	THF	Conventional	40	2.5	63
6	Neat	Conventional	40	3	53
7	Neat	MW	Not detected	10 min	<30
8	Basic alumina	MW	Not detected	10 min	<30

^a^ Isolated yields after column chromatography.

Having established the reaction conditions, various acyl chlorides were examined in this procedure to investigate the reaction scope, and several representative examples are summarized in Table 2. The reaction was clean and proceeded smoothly to give the corresponding 3-acylpyrroles in good to excellent yields. There were no remarkable differences in yields and reaction times between aromatic and aliphatic acyl halides in this procedure.

**Table 2:** One-pot synthesis of 3-acylpyrroles.

Entry	Product	Time (h)	Yield^a^(%)
1	**4a**	2.5	81
2	**4b**	3	83
3	**4c**	3	73
4	**4d**	3.5	78
5	**4e**	2.5	76

^a^ Isolated yields after column chromatography.

The structures of compounds **4a–4e** were deduced from their elemental analyses and their IR, ^1^H- NMR, and ^13^C-NMR spectroscopic data and no side products were identified. For example, the ^1^H- NMR spectrum of **4a** exhibited one singlet identified as the *N*-methyl moiety (δ 3.50), phenyl proton multiplets (δ 7.30, 3H and δ 7.41, 2H), along with multiplets [δ 7.51, 1H, δ 7.60, 1H and δ 7.81, 1H), for the pyrrole protons. The ^1^H-decoupled ^13^C-NMR spectrum of **4a** showed 10 distinct resonances that confirm the proposed structure. The IR spectrum of **4a** displayed a characteristic carbonyl (1,732 cm^-1^) stretching vibration.

Although the mechanistic details of the reaction are not known, a plausible rationalization may be advanced to explain the product formation. Presumably, the enamine **5** formed from *N*-methyl-hydroxylamine and DMAD attacked to acyl chloride to furnish intermediate **6**, which is converted to intermediate **7**. Subsequent reaction of **7 **with DMAD yields intermediate **8**, which undergoes an intramolecular cyclization reaction to generate **9**. Finally, products **4a-e** were formed from intermediate **9**.

**Scheme 2:**
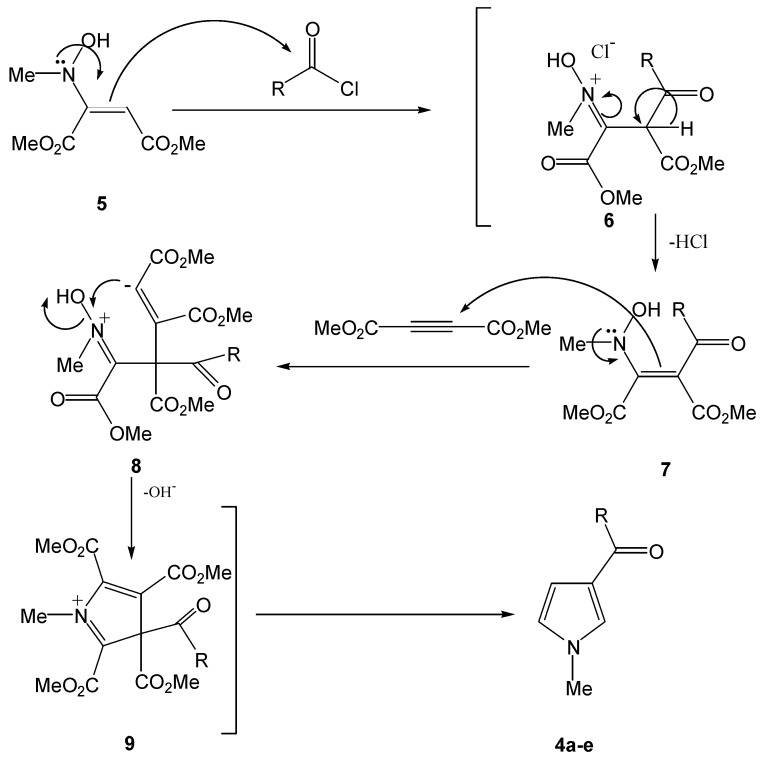
Plausible mechanism for the formation of the products **4a-e**.

## 3. Experimental

### 3.1 General

All reagents were purchased from Merck and used without further purification. ^1^H- and ^13^C-NMR spectra were recorded on a Bruker Avance AC-400 MHz instrument using CDCl_3_ as the deuterated solvent and TMS as internal standard. Elemental analyses were carried out on a Perkin-Elmer 240C elemental analyzer and are reported in percent atomic abundance. All melting points are uncorrected and measured in open glass-capillaries using Stuart melting point apparatus. Microwave experiments were conducted in a Milestone MicroSYNTH apparatus.

### 3.2. General procedure for preparation of 3-acylpyrroles ***4a-e***

Dimethylacethylene dicarboxylate (2 mmol) was added to a stirred solution of *N*-methyhydroylamine hydrochloride (2 mmol) and KHCO_3_ (3 mmol) in anhydrous CH_2_Cl_2_ (10 mL). The mixture was stirred for 15 min, and then a solution of an acyl chloride (2 mmol) and dimethylacethylene dicarboxylate (2 mmol) in CH_2_Cl_2_ (5 mL) was added slowly at 40 ºC. After completion of the reaction (2.5–3.5 h) as indicated by TLC on silica gel F_254_ (*n*-hexane/EtOAc 6:1), the solvent from the reaction mixture was evaporated under reduced pressure to leave a residue that was purified by column chromatography (*n*-hexane/EtOAc 6:1) to afford pure desired products **4a-e**.

*N-Methyl-3-benzoyl pyrrole* (**4a)**: IR (KBr) (ν_max, _cm^-1^): 1732 (C=O); ^1^H-NMR δ_H_ (ppm): 7.81 (m, 1H), 7.60 (m, 1H), 7.51 (m, 1H), 7.41 (m, Ph-H, 2H), 7.30 (m, Ph-H, 3H), 3.50 (s, 3H, N-Me); ^13^C-NMR δ_C_ (ppm): 163.32, 133.14, 132.24, 129.92, 128.77, 127.62, 127.10, 126.84, 125.75, 36.24; Anal. Calcd (%) for C_12_H_11_NO: C, 77.81; H, 5.99; N, 7.56. Found (%): C, 77.90; H, 5.91; N, 7.48.

*N-Methyl-3-acetyl pyrrole* (**4b**): IR (KBr) (ν_max, _cm^-1^): 1727 (C=O); ^1^H-NMR δ_H_ (ppm): 7.75 (m, 1H), 7.48-7.55 (m, 2H), 3.53 (s, 3H, N-Me), 2.09 (s, 3H, COMe); ^13^C-NMR δ_C_ (ppm): 160.22, 131.33, 129.12, 126.12, 124.78, 33.45, 27.50; Anal. Calcd (%) for C_7_H_9_NO: C, 68.27; H, 7.37; N, 11.37. Found (%): C, 69.05; H, 7.29; N, 11.31.

*N-Methyl-3-(4-chlorobenzoyl)-pyrrole* (**4c**): IR (KBr) (ν_max, _cm^-1^): 1720 (C=O); ^1^H-NMR δ_H_ (ppm): 7.77 (m, 1H), 7.51-7.58 (m, 2H), 7.38 (d, J=7.56Hz, 2H), 7.31 (d, J=7.56Hz, 2H), 3.47 (s, 3H, N-Me); ^13^C-NMR δ_C_ (ppm): 164.56, 135.66, 134.32, 131.11, 129.32, 128.09, 127.99, 126.25, 126.15, 33.36; Anal. Calcd (%) for C_12_H_10_ClNO: C, 65.61; H, 4.59; N, 6.38. Found (%): C, 65.65; H, 4.53; N, 6.34.

*N-Methyl-3-(2-methoxybenzoyl)-pyrrole* (**4d**): IR (KBr) (ν_max, _cm^-1^): 1721 (C=O); ^1^H-NMR δ_H_ (ppm): 7.83 (m, 1H), 7.49-7.54 (m, 2H), 7.31-7.38 (m, Ph-H, 4H), 3.87 (s, 3H, OMe), 3.50 (s, 3H, N-Me); ^13^C-NMR δ_C_ (ppm): 162.21, 137.62, 136.54, 135.66, 134.10, 133.22, 132.20, 130.34, 129.79, 127.40, 126.15, 67.71, 34.16; Anal. Calcd (%) for C_13_H_13_NO_2_: C, 72.54; H, 6.09; N, 6.51. Found (%): C, 72.58; H, 5.98; N, 6.48.

*N-Methyl-3-(4-nitrobenzoyl)-pyrrole* (**4e**): IR (KBr) (ν_max, _cm^-1^): 1717 (C=O); ^1^H-NMR δ_H_ (ppm): 7.70 (m, 1H), 7.66 (d, J=7.87Hz, 2H), 7.45-7.53 (m, 2H), 7.37 (d, J=7.87Hz, 2H), 3.54 (s, 3H, N-Me); ^13^C-NMR δ_C_ (ppm): 160.06, 133.06, 133.02, 131.21, 129.70, 128.56, 127.65, 127.05, 126.55, 37.09; Anal. Calcd (%) for C_12_H_10_N_2_O_3_: C, 62.60; H, 4.38; N, 12.17. Found (%): C, 62.80; H, 4.35; N, 12.08.

## 4. Conclusions

In summary, we have described a highly efficient one-pot procedure for the preparation of *N*-methyl-3-acylpyrroles by a three component reaction in CH_2_Cl_2 _using KHCO_3_ as catalyst. The reaction products were prepared in moderate to good yield, even with different substituted acyl halides. We believe that this procedure provides a valuable addition to current methodologies.
